# Dihydropyrimidine Dehydrogenase Polymorphism c.2194G>A Screening Is a Useful Tool for Decreasing Gastrointestinal and Hematological Adverse Drug Reaction Risk in Fluoropyrimidine-Treated Patients

**DOI:** 10.3390/cimb46090584

**Published:** 2024-09-04

**Authors:** Alessio Ardizzone, Maria Bulzomì, Fabiola De Luca, Nicola Silvestris, Emanuela Esposito, Anna Paola Capra

**Affiliations:** 1 Department of Chemical, Biological, Pharmaceutical and Environmental Sciences, University of Messina, Viale Ferdinando Stagno D’Alcontres, 31, 98166 Messina, Italy; aleardizzone@unime.it (A.A.); maria.bulzomi@unime.it (M.B.); fabiola.deluca@unime.it (F.D.L.); annapaola.capra@unime.it (A.P.C.); 2 Medical Oncology Unit, Department of Human Pathology “G. Barresi”, University of Messina, 98125 Messina, Italy; nicola.silvestris@unime.it; 3 Genetics and Pharmacogenetics Unit, “Gaetano Martino” University Hospital, Via Consolare Valeria 1, 98125 Messina, Italy

**Keywords:** DPYD polymorphisms, adverse drug reaction (ADR), fluoropyrimidines, 5-Fluorouracil (5-FU)

## Abstract

Although the risk of fluoropyrimidine toxicity may be decreased by identifying poor metabolizers with a preemptive dihydropyrimidine dehydrogenase (*DPYD*) test, following international standards, many patients with wild-type (WT) genotypes for classic variations may still exhibit adverse drug reactions (ADRs). Therefore, the safety of fluoropyrimidine therapy could be improved by identifying new *DPYD* polymorphisms associated with ADRs. This study was carried out to assess whether testing for the underestimated c.2194G>A (DPYD*6 polymorphism, rs1801160) is useful, in addition to other well-known variants, in reducing the risk of ADRs in patients undergoing chemotherapy treatment. This retrospective study included 132 patients treated with fluoropyrimidine-containing regimens who experienced ADRs such as gastrointestinal, dermatological, hematological, and neurological. All subjects were screened for *DPYD* variants DPYD2A (IVS14+1G>A, c.1905+1G>A, rs3918290), DPYD13 (c.1679T>G, rs55886062), c.2846A>T (rs67376798), c.1236G>A (rs56038477), and c.2194G>A by real-time polymerase chain reaction (RT-PCR). In this cohort, the heterozygous c.2194G>A variant was present in 26 patients, while 106 individuals were WT; both subgroups were compared for the incidence of ADRs. This assessment revealed a high incidence of gastrointestinal and hematological ADRs in DPYD6 carriers compared to WT. Moreover, we have shown a higher prevalence of ADRs in females compared to males when stratifying c.2194G>A carrier individuals. Considering that c.2194G>A was linked to clinically relevant ADRs, we suggest that this variant should also be assessed preventively to reduce the risk of fluoropyrimidine-related ADRs.

## 1. Introduction

The lifetime risk of cancer is likely due to changing lifestyles, habits, and increased life expectancy [1], in addition to genetic and environmental factors that can promote the formation of atypical cells characterized by autonomous, purposeless, and progressive growth [2].

Cancer therapies include surgery, radiotherapy, chemotherapy [3], and more recently also the use of immunotherapies, biological agents, and target therapies [4]. In this study, our attention focused on 5-Fluorouracil (5-FU), one of the most widely used anticancer drugs for the treatment of numerous solid tumors, including breast, colorectal, head, and neck cancers [5].

The most comprehensively investigated and well-recognized mechanism of 5-FU is its suppression of thymidylate synthase (TS) by fluorodeoxyuridylate (FdUMP), although many biochemical roles of this molecule remain poorly understood [6].

Clinical studies reported that the 5-year survival rate of patients with colorectal cancer who received 5-FU implants for chemotherapy was 56.12%, which was significantly better than the control group [7]. The 5-year disease-free survival rate was 48.98% in the 5-FU implants group and 34.62% in the control group [7]. However, despite 5-FU clinical benefits in treating several cancer forms, the use of this drug, or prodrugs like capecitabine, poses a clinical challenge as it can cause significant adverse drug reactions (ADRs) [8]. Indeed, 5-FU toxicity can affect many different organs and systems, occurring within a few days after therapy begins. Anorexia, nausea, and vomiting are typically the initial gastrointestinal symptoms, followed by mucositis and diarrhea [9]. In addition, 5-FU therapy, particularly when given as an intravenous bolus, can result in hematopoietic system disorders causing myelosuppression with leukopenia and thrombocytopenia, as well as in rare neurotoxicity [10,11]. These conditions favor the patient’s susceptibility to infections and hemorrhages, raising the risk of potentially fatal outcomes.

Thus, the discovery of biomarkers predictive of drug-related toxicities is crucial to preventing harmful conditions in patients undergoing chemotherapy with 5-FU. Numerous enzymes are involved in the metabolism of fluoropyrimidines, and many of these have intermediate metabolites [12]. Indeed, 5-FU is metabolized primarily in the liver through a series of biochemical processes that significantly influence its efficacy and toxicity [13]. In detail, after administration, 5-FU is converted into its active metabolite, 5-fluoro-deoxyuridine monophosphate (FdUMP), which inhibits thymidylate synthase, a crucial enzyme for DNA synthesis and exerts cytotoxic effects on rapidly dividing cancer cells [14]. This conversion is facilitated by the enzyme dihydropyrimidine dehydrogenase (DPD), which also plays a key role in the subsequent degradation of 5-FU to inactive metabolites, such as 5-fluoro-uracil (FUra) [15]. Additional enzymes, including dihydropyrimidinase (DYP) and uracil phosphoribosyltransferase (UPRT), further process these metabolites [16]. The metabolic pathways of 5-FU are complex and include the formation of several byproducts that are eventually excreted in the urine [17].

Another active metabolite, 5-fluorouridine triphosphate (FUTP), is incorporated into RNA, interfering with RNA processing and function, which contributes to the cytotoxic effects of 5-FU [18]. Additionally, 5-fluorodeoxyuridine triphosphate (FdUTP) is incorporated into DNA, causing damage to DNA and cell death [19].

Genetic variations associated with altered DPD enzyme activity affect drug metabolism and increase the risk of severe toxicity [20]. The activity of 5-FU is also modulated by co-administered drugs, such as leucovorin, which enhances its efficacy by stabilizing the 5-FU-thymidylate synthase complex [21]. Understanding these metabolic processes is essential for optimizing treatment regimens and managing potential side effects in patients undergoing 5-FU therapy [20].

Nevertheless, DPD is the rate-limiting step in this process, since it converts at least 80% of the amount of 5-FU or capecitabine that is delivered into 5-fluoro-5,6-dihydrouracil (5-FDHU) [22]. Dihydrofluorouracil is further metabolized to 5-fluoro-ureidopropionic acid (FUPA) and subsequently to alpha-fluoro-beta-alanine (FBAL), both of which are excreted in the urine and are generally considered inactive [23].

Consequently, in the presence of inactive or reduced DPD, the anabolic activation of 5-FU increases [24], leading to 5-FU-related ADRs [25].

The dihydropyrimidine dehydrogenase gene (*DPYD*) is located in 1p21.3; it contains 23 exons spanning about 950 kb [26]. It is a highly polymorphic gene, with several *DPYD* variants identified throughout the gene [27].

Currently, there are four well-known and clinically relevant *DPYD* variants: DPYD*2A (c.1905+1G>A, IVS14+1G>A), DPYD*13 (c.1679T>G), c.2846A>T, and c.1236G>A (in linkage disequilibrium with c.1129-5923C>G) [28]. Those have been strongly linked to partial or total loss of enzymatic activity and severe ADRs [29,30].

The most well-known variant is DPYD*2A (c.1905+1G>A, rs3918290), which causes a splice-site mutation leading to a non-functional enzyme and is associated with a high risk of severe toxicity [31]. Another important variant is DPYD*13 (c.1679T>G, rs55886062), which results in an amino acid change (I560S) and significantly reduces enzyme activity [32]. The c.2846A>T (rs67376798) variant leads to an amino acid substitution (D949V) and has been associated with reduced DPD activity and increased risk of 5-FU toxicity [33].

Also, the c.1236G>A (rs56038477) variant, often found in linkage disequilibrium with a haplotype containing other risk alleles, can also contribute to decreased enzyme function [34]. The c.2194G>A (rs1801160) variant results in a V732I amino acid substitution, which has been less consistently associated with toxicity but is still considered a variant of interest in pharmacogenetic testing [35].

Thus, screening for these *DPYD* variants before initiating 5-FU therapy can help identify patients at risk for severe toxicity and guide appropriate dose adjustments to improve safety and efficacy. Understanding the genetic landscape of *DPYD* variants is crucial for personalized medicine approaches in cancer treatment.

Although many genetic DPD variants do not impair enzyme activity or are still under investigation to better understand their functional impact, numerous attempts have been made to determine the best cost and time-effective method for assessing DPD deficiency and avoiding toxicity. However, the issue remains controversial as conflicting recommendations regarding the pre-emptive *DPYD* analysis in clinical practice have been released [36,37,38]. Other factors may be responsible for toxicity, such as a patient’s age, general condition, co-morbidities, as well as polymorphisms of other genes [39].

In 2019, the Italian Society of Pharmacology (SIF) together with the Italian Society of Medical Oncology (AIOM) published their recommendations on *DPYD* genotyping and prescription of fluoropyrimidines, stating that genotyping of the four core variants (c.1905+1G>A, c.1679T>G, c.2846A>T, and c.1236G>A) is considered mandatory, and the genotyping of additional variants is recommended in case of persistence of toxicity.

The non-synonymous variant c.2194G>A, despite being relatively common in our population (allele frequency reported in gnomAD: 0.04675), still lacks clear clinical interpretation, and controversial data about the outcomes associated with ADRs from fluoropyrimidine-based therapy have been provided [27].

Therefore, this study aimed to evaluate the clinical significance of c.2194G>A (DPYD*6; rs1801160) polymorphism to provide additional evidence about its association with the risk of ADRs related to a 5-FU regimen in a cohort of patients from Policlinic G. Martino Hospital of Messina.

## 2. Materials and Methods

### 2.1. Study Design and Patients

This retrospective study involved 132 adult oncologic patients. To mitigate the risk of severe fluoropyrimidine-induced toxicity, a pharmacogenetic pre-emptive test was conducted to determine the *DPYD* genotype and guide dose adjustments based on Italian recommendations. The study was approved in accordance with the Helsinki Declaration, and written informed consent was obtained from all participants. Between March 2022 and December 2023, blood samples were collected at the Genetics and Pharmacogenetics Unit of the “G. Martino” University Hospital of Messina. Genomic DNA was extracted from peripheral blood using the QIAamp Blood Kit (Qiagen GmbH, Hilden, Germany), following the manufacturer’s protocol. DNA concentration was measured using a Qubit 2.0 fluorimeter with the Qubit dsDNA BR Assay kit, as recommended by the manufacturer. *DPYD* variants analyzed included: DPYD*2A (IVS14+1G>A, c.1905+1G>A, rs3918290), DPYD*13 (c.1679T>G, rs55886062), c.2846A>T (rs67376798), c.1236G>A (rs56038477), and c.2194G>A (rs1801160). These were analyzed using Real-Time Polymerase Chain Reaction (RT-PCR) (QuantStudio3, Applied Biosystems, Waltham, MA, USA) with genotyping TaqMan assays provided in the diagnostic kit (AMPLI set DPYD Real Time, Dia-chem S.r.l., Napoli, Italy) following the specified protocol. Control samples supplied by the kit with known genotypes: wild-type homozygotes, heterozygotes, and mutated homozygotes for each *DPYD* variant, and negative control, no DNA, were always included in the analysis session. The amplification protocol takes approximately two hours, with 1 cycle at 95 °C for 10 min, and at 92 °C for 15 s and at 60 °C for 90 s (step of detection) repeated for 45 cycles. The tests are carried out by a specific program (Allelic Discrimination) of the real-time PCR tool previously set with the different JOE/VIC and FAM primer–probe associated with each variant in analysis with ROX, like reference dye. Furthermore, the amplification graph plot was also analyzed to check the positive and negative controls and to make sure that the amplification took place in the mode correctly in each sample.

All collected data were compiled into a database that included information on *DPYD* genotypes, clinical data, drug dose adjustments, and reported adverse drug reactions. The database was used to review patient medical records and document episodes of toxicity occurring during the first two treatment cycles.

The observed frequencies of *DPYD* variants were compared with the reported general frequencies present in the Genome Aggregation Database (gnomAD).

### 2.2. Statistical Analysis

The values are shown as the mean ± standard deviation (SD) of N observations, where N is the number of individuals involved in the study. The association between the DPYD*6 variant, WT, and ADRs was evaluated by chi-square (χ^2^) test using GraphPad Prism version 8.0 (La Jolla, CA, USA). Only findings with a *p*-value less than 0.05 were considered statistically significant. All methods were conducted under relevant guidelines and regulations.

## 3. Results

### 3.1. Characteristics of Patient Candidates for Fluoropyrimidine Therapies

A total of 132 patients treated with 5-FU or its prodrugs were evaluated in the study; 72 (54.55%) patients were males and 60 (45.45%) were females, and the mean age of the total cohort was 66.5 ± 10.4 years. All subjects were Caucasian and presented three different types of cancer: colorectal, gastric, or breast. In particular, 91 individuals were affected by colorectal cancer (68.94%), 37 suffered from gastric cancer (28.03%), and 4 from breast cancer (3.03%). Moreover, in the analyzed cohort, part of the subjects (26.52%) were affected by comorbidities with a higher prevalence of type 2 diabetes and hypertension, while 73.48% were not affected by any diseases.

The description of the cohort is reported in Table 1.

### 3.2. Analysis of DPYD Polymorphisms in the Cohort

The *DPYD* variant c.2194G>A (rs1801160) was evaluated in a cohort of subjects resulted WT for the DPYD*2A (IVS14+1G>A, c.1905+1G>A, rs3918290), DPYD*13 (c.1679T>G, rs55886062), c.2846A>T (rs67376798), and c.1236G>A (rs56038477). In patients also receiving combined treatment with irinotecan, the presence of the pharmacogenetic variant UGT1A1*28 (rs3064744) was excluded. All the information about c.2194G>A polymorphism are summarized in Table 2.

**Table 2:** Variant c.2194G>A tested in the DPYD gene in the cohort of patients.

Variant	rsID	Nucleotide Change	Amino Acid Change	Dpd Activity	*DPYD* Carriers (n) (%)	Suggested Dose (5-FU/ Capecitabine etc.)	Allele Frequency Reported in gnomAD	Allele Frequency Observed in the Cohort
*6	rs1801160	c.2194G>A	(p.Val732Ile)	Normal function	26 (19.70%)	100% ab initio, 85% in case of toxicity	0.04675	0.065

### 3.3. Reported ADRs after Fluoropyrimidines Treatment in Carrier Patients of WT and DPYD*6

Regarding ADRs, they were categorized into four main groups: gastrointestinal (76.5%), dermatological (8.33%), hematological (52.27%), and neurological (1.52%). Gastrointestinal ADRs were the most common in the cohort, including nausea or vomiting (18.18%), diarrhea (42.41%), and stomatitis (15.91%). Hematological ADRs were also quite frequent, comprising fever (4.55%), leukopenia (14.39%), neutropenia (21.21%), anemia (5.30%), and thrombocytopenia (6.82%). Dermatological ADRs primarily involved hand-foot syndrome, with a prevalence of 8.33%, while neurological ADRs were less common, occurring in only 1.52% of patients. ADRs documented in the cohort are described in Table 3.

**Table 3:** ADRs were reported in the cohort of patients treated with fluoropyrimidines.

ADRs Gastrointestinal (76.5%)	Grade ≥ 2
Nausea/Vomiting	24 (18.18%)
Diarrhea	56 (42.41%)
Stomatitis	21 (15.91%)
**ADRs Dermatological** (**8.33%**)	**Grade ≥ 2**
Hand–foot syndrome	11 (8.33%)
**ADRs Hematological** (**52.27%**)	**Grade ≥ 3**
Fever	6 (4.55%)
Leucopenia	19 (14.39%)
Neutropenia	28 (21.21%)
Anemia	7 (5.30%)
Thrombocytopenia	9 (6.82%)
**ADRs Neurological** (**2.94%**)	
Peripheral neuropathy	4 (2.94%)

To better understand the association between the DPYD*6 polymorphism and the manifestation of ADRs following treatment with fluoropyrimidines, we divided the cohort into two subgroups based on their genotype: wild-type (WT) and c.2194G>A carriers. Regarding gastrointestinal ADRs, we observed significant differences in the incidence of nausea/vomiting, diarrhea, and stomatitis between WT and DPYD*6 variant carriers. However, no significant differences were found between the two groups for dermatological ADRs, specifically hand–foot syndrome.

For hematological adverse events, the presence of c.2194G>A was associated with increased incidences of leucopenia, neutropenia, and thrombocytopenia. No substantial statistical differences were observed for fever or anemia. Additionally, no significant differences in the rate of peripheral neuropathy were found between the two subgroups, as shown in Table 4.

### 3.4. Assessment of ADRs between Patients with Mutated DPYD*6 and WT Stratifying by Gender

According to the literature research, specific gender differences in the c.2194G>A carrier subjects have not been well established. Therefore, we stratified the previously identified statistically significant ADR comparisons by gender to determine potential differences between male and female DPYD*6 variant carriers compared to WT individuals.

**Figure 1:**
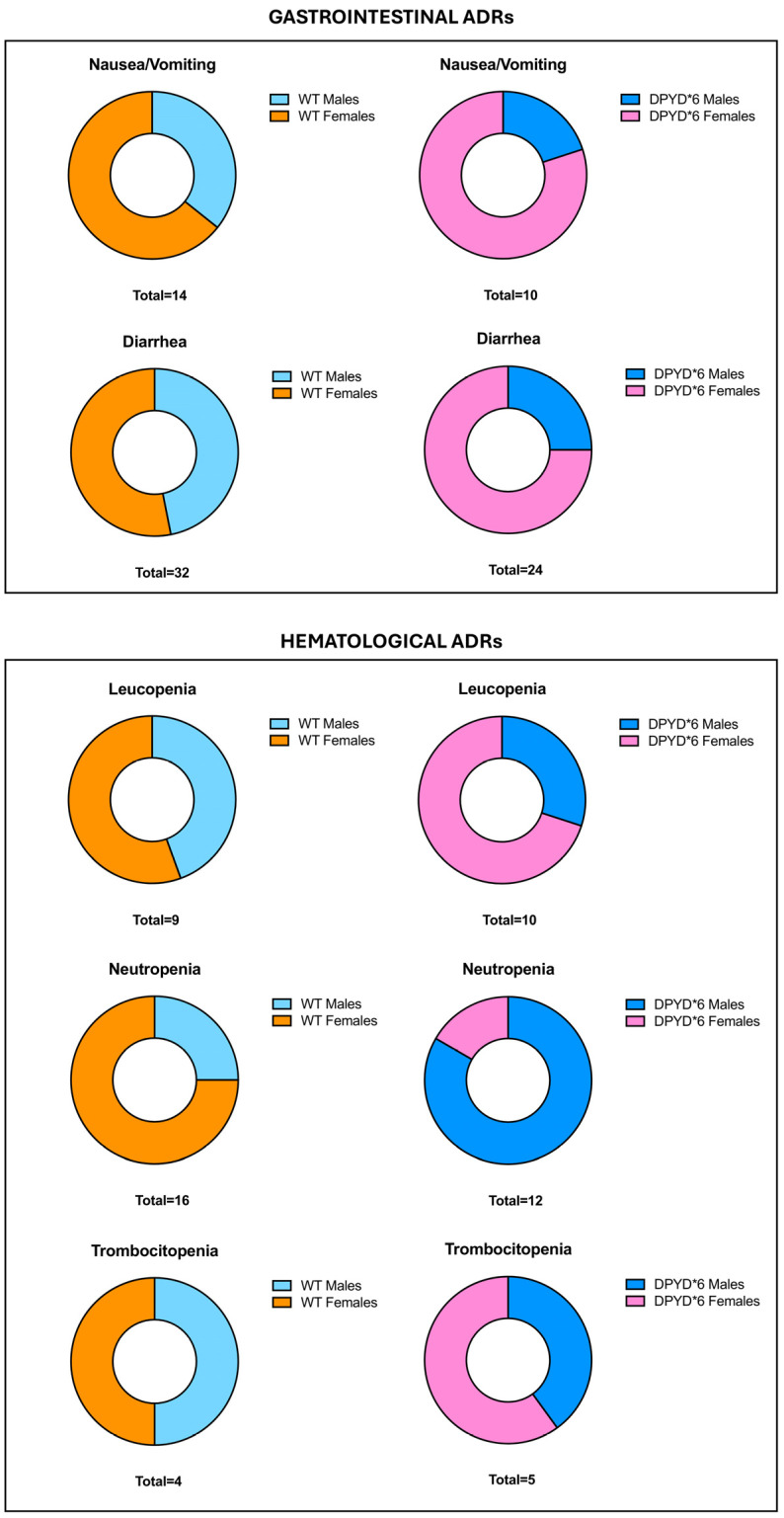
Evaluation of ADRs stratifying subjects with mutated DPYD*6 by gender.

This evaluation revealed that nausea/vomiting, diarrhea, leucopenia, and thrombocytopenia were more prevalent in females presenting c.2194G>A than in males’ carriers, also compared to the gender stratification performed on WT (Figure 1). Differently, neutropenia was more increased in male subject carriers of DPYD*6 compared to females, while an opposite trend was detected in WT individuals, in which neutropenia was more prevalent in females than in males (Figure 1).

## 4. Discussion

The goal of personalized medicine is to tailor the therapeutic options according to the individual and unique clinical and molecular profile of each patient [40,41]. Determining the relationship between a patient’s biological traits and the drugs’ effects is essential for identifying predictive biomarkers and personalizing treatment dosages [42,43]. In this frame, precision medicine has made great progress thanks to the research of genetic polymorphisms [44], which has proven extremely useful for both evaluating therapy outcomes and ensuring drug safety [45]. Indeed, identifying genetic variants in patients can help in selecting the appropriate chemotherapy drug and dosage [35,46].

Thus, a crucial first step toward customized medicine is making the correct pharmaceutical selection based on a patient’s genetic profile [47]. As a consequence, since single nucleotide polymorphisms (SNPs) increase the patient’s ADR risk, their pre-emptive detection can aid in tailoring treatment approaches [48,49].

The main aim of this investigation was to study the metabolism of fluoropyrimidines in patients with the DPYD*6 polymorphism and to clarify the role of this SNP in the risk of ADRs. This issue is critical as the anabolic and catabolic processes of fluoropyrimidines exhibit considerable interindividual variability, which significantly affects clinical responses to therapy. The missense variant c.2194G>A (rs1801160), characterized by the substitution of valine with isoleucine at position 732 (p.Val732Ile), remains a topic of debated clinical interpretation. Some studies did not associate the presence of c.2194G>A in the occurrence of fluoropyrimidine toxicity [26,50] and in vitro tests demonstrated a normal enzyme activity [51]. For the well-known mutations (DPYD*2A IVS14+1G>A, c.1905+1G>A, DPYD*13 (c.1679T>G), c.2846A>T, and c.1236G>A), several clinical data show a clear association with toxicities, while variants like c.2194G>A still lack evident clinical outcomes; instead, heterogenous data are provided [27].

Indeed, despite that international guidelines recommend fluoropyrimidine dose adjusted to genotype-predicted DPD activity based on the four variants (rs3918290, rs55886062, rs67376798, and rs56038477), other polymorphisms can be associated with the development of ADRs.

For instance, Božina and colleagues assessed the relationship between three additional *DPYD* polymorphisms: c.496A>G (rs2297595), *6 c.2194G>A (rs1801160), and *9A c.85T>C (rs1801265) and severe ADR risk [28]. From this study’s results, both *DPYD* c.496A>G and c.2194G>A variants were suggested to be considered for inclusion in the *DPYD* genotyping panel since they were strongly related to gastrointestinal and hematological side effects [28].

In the present study, the obtained data also showed a higher ADR incidence in DPYD*6 mutated subjects compared to WT, especially regarding the gastrointestinal and hematological side effects. This finding confirms that, among *DPYD* polymorphisms, DPYD*6 also plays a significant role in fluoropyrimidine toxicity. National recommendations advocate for including this SNP as an “additional” variant to analyze in cases of toxicity during treatment, whereas international guidelines consider its utility in clinical practice to be limited.

The total frequency of this variant in the general population, as reported on the Genome Aggregation Database (gnomAD), is 0.04531. In our cohort, this variant was found with a frequency of 0.065, which is slightly higher than that reported in reference databases.

In this cohort, most ADRs were predominantly associated with the female subgroup of patients. This observation aligns with the existing literature, which suggests that women experience 30–50% more ADRs than men. The primary reasons for this gender difference are attributed to factors such as polypharmacy, sex-related differences in pharmacokinetics or pharmacodynamics, and potential variations in pharmacogenomic profiles [52].

Furthermore, when stratifying c.2194G>A carrier subjects according to gender, a greater prevalence of gastrointestinal ADRs was found in women than in men. The only exception was neutropenia, which was more common in male DPYD*6 carriers than in female carriers.

This evidence certainly needs further validation, since differences in body composition, hormonal influences, and enzyme activity can all impact drug metabolism and response. For instance, by analyzing in-depth possible hormonal influences on the DPYD*6 polymorphism, fluoropyrimidine metabolism, and potential ADR risks.

Hence, the results obtained from the present study corroborate a major occurrence of gastrointestinal and hematological ADRs following the start of the fluoropyrimidine chemotherapeutic regimen when DPYD*6 is present.

The appearance of these adverse effects led to a reduction in the dosage of fluoropyrimidine compared to the standard treatment carried out on WT subjects. According to the AIOM/SIF recommendations, for patients heterozygous for the c.2194G>A, a reduction in the drug dose is not necessary at the beginning of treatment, but a 15% reduction is recommended in case of toxicity.

In accordance with this, in our study, a 15% dose reduction of fluoropyrimidines was suggested for patients with the c.2194G>A mutation; in some cases, considering various factors including ADRs, the advanced age of the patient, the polypharmacy, and various comorbidities, the dose was also reduced by 20/25%. In the examined cohort, however, 45.45% of patients with WT had a dose reduction of fluoropyrimidines of approximately 10–15%.

The main limitation of this study is its retrospective nature. Moreover, while the role of c.2194G>A appears significant, this result may have been affected by the small number of carriers, particularly concerning treatment tolerability in association with some ADRs (hematological, neurological). Additionally, the cohort consisting solely of Caucasians of European origin limits the generalizability of the findings, as other populations were not considered. The varying frequencies of relevant pharmacogenetic variants in different ethnic groups is also an issue that needs to be addressed.

For example, a recent publication confirmed that African populations have markedly distinct allele frequencies in genes involved in the absorption, distribution, metabolism, and excretion of drugs, compared with Caucasians and Asians [53].

We included patients with a slight heterogeneity of clinical baseline conditions and treated with various protocols containing fluoropyrimidines independently or combined with other drugs or immunotherapy to assess the impact of DPYD*6 polymorphism.

The *DPYD* test is still not universally accepted; although it has been demonstrated to be cost-effective, several subjects are examined only after an ADR has occurred, thus abolishing the advantage of preemptive genotyping and reducing the safety of fluoropyrimidine therapies [27]. Especially because another recent publication supported the therapeutic efficacy of treatments at the proposed dose adjusted based on the pharmacogenetic tests in a manner comparable to patients with WT [54].

Certainly, soon, advanced genomic approaches, available and economically sustainable, will broaden the scenario to new genotype–drug interactions, which will certainly have an important clinical impact, to more personalized and safer drug therapy [55].

We must underline that additional variants in the *DPYD* gene may also affect enzymatic activity, leading to the accumulation of fluoropyrimidines and increased toxicity. Furthermore, other genes involved in the metabolism of these drugs could be implicated. These pieces of information, combined with the results of this study, in which fluoropyrimidine-associated ADRs were likely attributable to the presence of the heterozygous c.2194G>A variant, suggest that the analysis of this polymorphism should not be underestimated. Therefore, we retain that identifying and confirming this variant through routine clinical tests before the start of chemotherapy treatments could be crucial in preserving patient health and reducing the risk of severe drug-related events.

Furthermore, while our research indicates an association between the c.2194G>A variant and ADRs, we acknowledge that it did not directly assess the impact of this variant on 5-FU pharmacokinetics. In fact, our study primarily focused on correlating the variant with the incidence of ADRs, and we did not include pharmacokinetic data to explore how the c.2194G>A variant might influence 5-FU absorption, distribution, metabolism, and excretion. We assume that understanding the pharmacokinetic effects of this variant is crucial for a comprehensive assessment of its clinical significance in oncology practice. Thus, future research will be necessary to investigate the direct effects of the c.2194G>A variant on 5-FU pharmacokinetics, which will provide a more complete understanding of how this genetic variant impacts treatment outcomes.

Additionally, drug–drug interactions (DDIs) present a significant challenge in the context of fluoropyrimidine therapy, particularly for patients with the DPYD*6 polymorphism. Medications that inhibit or induce dihydropyrimidine dehydrogenase (DPD), the enzyme responsible for metabolizing fluoropyrimidines, can exacerbate or mitigate treatment-related toxicities. For instance, co-administration with DPD inhibitors or other drugs affecting gastrointestinal pH or motility can alter the efficacy and safety profile of fluoropyrimidines. Moreover, gender-specific differences in drug metabolism and interaction profiles may further complicate treatment, as evidenced by our study’s findings on ADRs. Therefore, careful consideration of potential DDIs is essential for optimizing therapeutic strategies since, as in the case of our study, patients are subjected to polytherapy. Personalized medicine approaches must integrate preemptive genotyping with comprehensive medication reviews to identify and manage interactions proactively, ensuring safer and more effective treatment outcomes. Future research should focus on understanding the interplay between genetic variants and drug interactions to refine dosage guidelines and improve patient care.

## 5. Conclusions

The results obtained in this study demonstrated a higher incidence of gastrointestinal and hematological ADRs in patients with the DPYD*6 variant compared to those who were WT. Notably, these adverse effects were more prevalent in female patients, except for neutropenia, which was more common in male carriers. The findings suggest that the DPYD*6 polymorphism significantly contributes to fluoropyrimidine toxicity, supporting its inclusion in genetic screening to tailor chemotherapy doses and improve patient safety.

Taken as a whole, the outcomes of the present study represent an important add-on and are consistent with other scientific evidence in the same research field. Indeed, Del Re and colleagues also indicated that the c.2194G>A variant is associated with clinically relevant ADRs in addition to the already known rs3918290, rs55886062, rs67376798, and rs56038477 variants [27]. Otherwise, our study also indicated a stronger ADRs incidence in c.2194G>A female subjects than males, thus opening a new perspective for future evaluations.

Furthermore, genotype-guided pharmacological treatment, as reported in the literature, significantly reduced the incidence of severe ADRs, and its application was found to be safe and feasible in various organizations and contexts of European healthcare [56]. Moreover, its long-term efficacy was also proved in a prospective multicenter study [54].

The success of antineoplastic treatments requires special attention and surveillance regarding related toxicities. Integrated pharmacogenetic approaches can support the true personalization of therapy and ensure a better quality of life for patients. It is essential to work in a multidisciplinary team that can interpret the data collected, consider comorbidities and polytherapy, and monitor the patient long-term, also providing large prospective multicenter studies. Further investigations are needed to conclusively confirm these preliminary findings and to provide more comprehensive data regarding the influence of the c.2194G>A variant on ADRs related to fluoropyrimidine administration.

## Figures and Tables

**Table 1:** Characteristics of the cohort of patients.

Characteristics	Cohort
Patients	132
Gender (M/F)	72/60
Age (years)	66.5 ± 10.4
Ancestry	Caucasian, European
Colorectal cancer	91 (68.94%)
Gastric cancer	37 (28.03%)
Breast cancer	4 (3.03%)
**Treatment**	
FU-LV (De Gramont regimen)	6 (4.5%)
5-FU	3 (2.3%)
FOLFOX	90 (68.2%)
FOLFIRI	14 (10.6%)
FOLFIRINOX	15 (11.4%)
Capecitabine	4 (3%)
**Combination with immunotherapy**	
Yes	30 (22.7%)
No	102 (77.3%)
**Comorbidity**	34 (25.75%)
**No Comorbidity**	98 (74.25%)

**Table 4:** DPYD*6 polymorphism c.2194G>A in associations with ADRs.

Side Effect	WT (*n* = 106)	DPYD*6 Polymorphism c.2194G>A (*n* = 26)	*p*-Value
**ADRs—Gastrointestinal**			
**Nausea/Vomiting** (24 cases)	14/106	10/26	*p* = 0.002
**Diarrhea** (56 cases)	32/106	24/26	*p* < 0.001
**Stomatitis** (21 cases)	15/106	6/26	*p* = 0.03
**ADRs—Dermatological**			
**Hand–foot syndrome** (11 cases)	8/106	3/26	*p* = 0.45
**ADRs—Hematological**			
**Fever** (6 cases)	3/106	3/26	*p* = 0.09
**Leucopenia** (19 cases)	9/106	10/26	*p* < 0.001
**Neutropenia** (28 cases)	16/106	12/26	*p* = 0.002
**Anemia** (7 cases)	5/106	2/26	*p* = 0.62
**Thrombocytopenia** (9 cases)	4/106	5/26	*p =* 0.01
**ADRs—Neurological**			
**Peripheral Neuropathy** (4 cases)	2/106	2/26	*p =* 0.17

## Data Availability

All the results were included in this study and are available to the corresponding author’s address.
